# A three-dimensionally preserved lobopodian from the Herefordshire (Silurian) Lagerstätte, UK

**DOI:** 10.1098/rsos.172101

**Published:** 2018-08-08

**Authors:** Derek J. Siveter, Derek E. G. Briggs, David J. Siveter, Mark D. Sutton, David Legg

**Affiliations:** 1 Earth Collections, University Museum of Natural History, Oxford OX1 3PW, UK; 2 Department of Earth Sciences, University of Oxford, Parks Road, Oxford OX1 3AN, UK; 3 Department of Geology and Geophysics and Yale Peabody Museum of Natural History, Yale University, PO Box 208109, New Haven, CT 06520-8109, USA; 4 School of Geography, Geology and the Environment, University of Leicester, Leicester LE1 7RH, UK; 5 Department of Earth Sciences and Engineering, Imperial College London, London SW7 2BP, UK; 6 School of Earth and Environmental Sciences, University of Manchester, Williamson Building, Oxford Road, Manchester M13 9PL, UK

**Keywords:** exceptional preservation, Herefordshire Lagerstätte, lobopodians, Onychophora, Panarthropoda, Silurian

## Abstract

The Herefordshire (Silurian) Lagerstätte (approx. 430 Myr BP) has yielded, among many exceptionally preserved invertebrates, a wide range of new genera belonging to crown-group Panarthropoda. Here, we increase this panarthropod diversity with the lobopodian *Thanahita distos*, a new total-group panarthropod genus and species. This new lobopodian preserves at least nine paired, long, slender appendages, the anterior two in the head region and the posterior seven representing trunk lobopods. The body ends in a short post-appendicular extension. Some of the trunk lobopods bear two claws, others a single claw. The body is covered by paired, tuft-like papillae. *Thanahita distos* joins only seven other known three-dimensionally preserved lobopodian or onychophoran (velvet worm) fossil specimens and is the first lobopodian to be formally described from the Silurian. Phylogenetic analysis recovered it, together with all described *Hallucigenia* species, in a sister-clade to crown-group panarthropods. Its placement in a redefined Hallucigeniidae, an iconic Cambrian clade, indicates the survival of this clade to Silurian times.

## 1. Introduction

**Figure 1.**
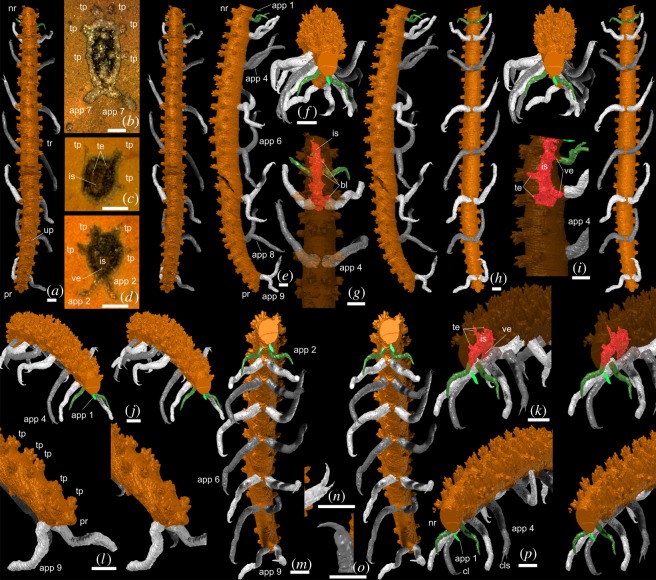
*Thanahita distos* gen. et sp. nov., virtual reconstructions (*a*,*e*–*p*) and specimen in the rock (*b*–*d*), OUMNH C.29699. (*a*,*e*,*f*,*h*,*j,m*) Whole specimen; dorsal, right lateral, frontal, ventral, anterior oblique, anterior ventral stereo pairs. (*b*) The specimen on the surface of the split nodule, before grinding. (*c*,*d*) The specimen shown on two individual ground surfaces between appendage 2 and 3 (slice 665) and at appendage 2 (slice 705), respectively, which are 0.8 mm apart. (*g*,*i*,*k*) Anterior part of specimen with external surface rendered transparent to show preserved internal structures; dorsal, right lateral, anterior oblique stereo pairs. (*l*) Posterior part of specimen, postero-lateral oblique stereo-pair. (*n*,*o*), Double (*n*) and single (*o*) claws on left fifth and left sixth appendages, respectively; sublateral views. (*p*) Anterior part of specimen; anterior oblique stereo-pair. Scale bars are all 1 mm. app 1–app 9, appendages 1–9; cl, claw; cls, claws; is, internal structures; nr, neck region; pr, post-appendicular region; te, tabular extension of internal structures towards tufted papilla; tp, tufted papilla; tr, trunk; up, unpaired papilla; ve, ventral extension of internal structures towards appendage.

The Herefordshire Lagerstätte from the Welsh Borderland, UK is remarkable in preserving the soft part morphology of a wide variety of marine invertebrates of mid-Silurian, Wenlock Series age (approx. 430 MYr) in three dimensions [1,2]. Since its discovery more than 20 years ago, the deposit has yielded a diversity of arthropods that have contributed much to our understanding of the palaeobiology and early history of the group ([3], and references therein). Here, we describe a new lobopodian, *Thanahita distos* gen. et sp. nov., the first from the Herefordshire fauna (figure 1).

**Figure 2.**
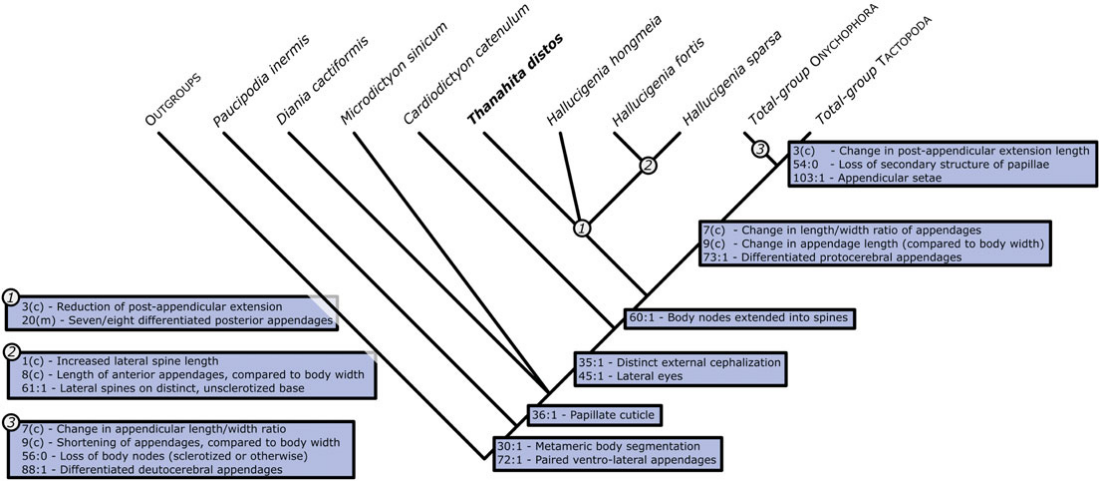
The phylogenetic position of *Thanahita distos* gen. et sp. nov. and the relationships of lobopodian-grade panarthropods indicated on a compacted tree, together with key synapomorphies. This represents a single most parsimonious tree with a score of 22.518, produced using the phylogenetic methodology outlined in §2, Material and methods. The full tree is given in the electronic supplementary material, figure S1. c, continuous; m, meristic.

Lobopodians are similar in morphology to extant terrestrial onychophorans (velvet worms), but many of these similarities are now recognized as plesiomorphies. Recent phylogenetic analyses (e.g. [4–8]) recovered a paraphyletic Lobopodia. Only a subset of lobopodians fall out as stem-onychophorans; others lie in the stem-groups of Euarthropoda, Tardigrada, or both (=Tactopoda; figure 2; electronic supplementary material, figure S1).

Lobopodians are rare in the fossil record except in the Cambrian, which has yielded 20 or more species (e.g. [9]). There are only two Ordovician records, both of undescribed species: from the Lower Ordovician (Floian), Upper Fezouata Formation of Morocco [10] and the uppermost Ordovician (Hirnantian) Soom Shale of South Africa [11]. An undescribed animal from the mid-Silurian (upper Llandovery/lower Wenlock series) Waukesha biota, Wisconsin, USA was initially reported as a ‘myriapod-like animal’ [12,13], but later regarded as closer to the lobopodians or stem-group arthropods [14]. A similar undescribed form is known from the Silurian Eramosa Lagerstätte, Ontario, Canada [15]. Two lobopodians are known from the Carboniferous Mazon Creek Fauna of Illinois, *Carbotubulus waloszeki* and *Helenodora inopinata* [16,17] (*Ilyodora divisa* and *Ilyodora elongata* from Mazon Creek are regarded as *nomina dubia* [17,18]). *Antennipatus montceauensis* from the Carboniferous Montceau-les-Mines biota in southern France may belong to stem or crown-group Onychophora [18].

Recent reassessments of the only three putative post-Palaeozoic onychophorans supported only *Cretoperipatus burmiticus* as an onychophoran, leaving the affinities of *Tertiapetus dominicanus* and *Succinipatopsis balticus* as uncertain ([18]; but see [19]). All come from amber, with *T*. *dominicanus* and *S*. *balticus* each being known from a single specimen and *C*. *burmiticus* from four specimens.

## 2. Material and methods

The only known specimen, preserved in a concretion, was serially ground at 20 µm intervals. Digital photographic capture of each surface produced a tomographic dataset that was rendered and studied as a three-dimensional virtual fossil using the SPIERS software suite [20,21]. The virtual fossil was interpreted using the on-screen rotational, variable magnification, stereoscopic-viewing and virtual dissection facilities of this suite, augmented by hard-copy stereoscopic images.

Phylogenetic analysis of the new species was undertaken using a dataset of 55 taxa and 128 characters (electronic supplementary material, text S1, §§2 and 3) with the TNT v. 1.5 program [22]. The dataset was based largely on that of Smith & Ortega-Hernández [5], with numerous modifications for the inclusion of continuous and meristic characters. Tree searches used traditional search options of 1000 replicates with TBR branch swapping, saving 100 trees per replicate; additional searches using ‘New Technology’ heuristics (with various settings) in all cases recovered the same most parsimonious tree (MPT) as the traditional search. Characters were weighted using implied weighting with a concavity constant of 3 [23]. Branch support values were obtained using 100 replicates of symmetric resampling, each consisting of traditional searches with TBR branch swapping, and a change probability of 33% [24]. Our analysis resulted in a single MPT (electronic supplementary material, figure S1) with a score of 22.518.

## 3. Systematic palaeontology

Panarthropoda Nielsen [25]

Family: Hallucigeniidae [26]

*Diagnosis*: Lobopodian panarthropods with ovoid head; a neck region bearing two or three pairs of non-annulated tentacle-like limbs that are thinner than the trunk lobopods; a trunk region bearing seven or eight long, tubular, smooth trunk lobopods, variously with either one or two claws; a short post-appendicular region that is less than half as long as the distance between the last two lobopod pairs or is absent; body bearing dorso-lateral pairs of spinose projections or papillae. (Revised from Caron & Aria [8]; see also below, Discussion, systematics.)

*Thanahita distos* gen. et sp. nov.

*Etymology*: From the Greek, *thea*, goddess, and Anahita (short form: Anita), the ancient Persian name of the Indo-Iranian deity of ‘the Waters’ (gender feminine)—named for Anita Siveter; and *distolos*, in pairs, plus *acherdos*, a hedge shrub, alluding to the bilateral tufted papillae on the trunk.

*Holotype* (and only known specimen): OUMNH C.29699 (figure 1*a*–*p*).

*Horizon and locality:* Upper part of Wenlock Series, Silurian, Herefordshire, UK.

*Generic and specific diagnosis*: A hallucigeniid with a body at least 15 times as long as wide, including a very short post-appendicular region. There are at least nine appendages, with at least two in the neck region and seven in the trunk. All appendages that are essentially complete (2–9) are relatively long and slender, with appendage 2 more slender and shorter than 3–9. Appendage 2 tapers distally to a point; two claws are present distally on appendages 4 and 5 and a single claw on appendages 6–9. Pairs of raised tufted cuticular papillae occur on the neck region and trunk: 23 immediately flank the median line dorsally and at least 17 smaller less regular ones occur more laterally.

*Description*: The incomplete end of the specimen (figure 1*j,n*) is interpreted as anterior because the spacing of successive appendages increases markedly posterior of appendage 3 (figure 1*e*), as it does in the transition from neck region to trunk in some other lobopodians (e.g. *Cardiodictyon* and *Hallucigenia*; e.g. [27]). The pronounced increase in size between appendages 2 and 3 may also reflect this transition, but size difference is less reliable as a distinguishing feature of the neck region. In addition, the short projection beyond the last appendage pair at the opposite end of the body is typical of the posterior termination in other lobopodians (e.g. *Luolishania*, *Hallucigenia*, *Diania*; see [9]).

The preserved length of the specimen (the anteriormost part of the head is missing) is 29.5 mm with a maximum width and depth of 1.9 mm and 2.3 mm, respectively. The body is more than 15 times longer than wide in dorsal view, widest just anterior to the mid-length and narrowing slightly anteriorly and posteriorly and is about one-third maximum width at the posterior extremity (figure 1*a*). It is oval in transverse section (figure 1*f*,*m*), and bears at least nine pairs of appendages. A short subconical region extends beyond the last pair of lobopods (figure 1*e*,*l*).

The appendages are attached to the body ventrally close to the trunk midline (figure 1*h*,*m*). They are similar in overall morphology, long and slender and tapering distally. Appendage 2 (in the neck region) is the shortest and most slender; appendages 3–9 (trunk appendages) have length to width ratios of 7 : 1 or 8 : 1. Appendage length increases posteriorly to a maximum in the sixth and seventh pair which are similar in length (estimated at 6.5 mm) and decreases beyond these. Only part of the left appendage of the first pair (neck region; figure 1*e*,*f*,*j*,*p*) was recovered and the morphology of this appendage is unknown. The second appendage (neck region; figure 1*e*,*j*,*p*) attaches about 1.0 mm behind the first; the left is incomplete, but the right terminates in a gradually tapering point. Appendage 3 is significantly longer than appendage 2 (figure 1*e*,*h*,*j*,*m*); the distal part was not recovered; it is attached about 1.7 mm farther from appendage 2 as 2 is from 1. Appendage 4 (figure 1*e*,*h*,*p*) and successive trunk lobopods are much more widely spaced. Appendages 4 and 5 terminate in a pair of short, curved claws, the inner longer than the outer (figure 1*h,j,m,n,p*). The outer claw is absent on appendage 6 and beyond (figure 1*e,h,m,o*).

Appendage pairs 1 to 4 are directed anteriorly. The left appendage of pairs 5 to 9 is also directed anteriorly, but the right appendage projects posteriorly (figure 1*a*,*e*,*j*,*h*,*m*). The direction of the curved claws conforms to the orientation of the appendages (they are not articulating podomeres, as occur in euarthropods, but sclerites: e.g. [5]).

Annulations have not been detected on the body or the appendages; if present, their spacing and relief are too fine to be resolved. The body bears subcircular (dorsal outline), tufted papillae (figure 1*a,b–e*,*f,m*,*p*) which extend above the dorsal trunk surface by about one-quarter to one-third its height. Each papilla comprises a boss-like base supporting some four to six short tapering projections, the central of which is often the most prominent. The papillae are largest dorsally (approx. 500 µm), where some 23 pairs immediately flank the midline (figure 1*a*,*e*). The spacing of these papilla pairs are somewhat inconsistent, and in places more dorsally positioned pairs lie in close proximity, making it difficult to relate them confidently to the appendage pairs. One pair of papillae corresponds in position to each appendage pair. An intervening pair of papillae is present between appendages 2 and 3, two pairs between appendages 3 and 9 and one beyond appendage 9 in the post-appendicular region. An extra unpaired papilla is present on the right side just posterior to appendage 8 (figure 1*a*). Rows of smaller and more widely spaced papillae are present dorsolaterally, and laterally, usually paired across the trunk, and there are a few small, muted paired papillae ventrally (figure 1*h*,*m*). The preservation of the papillae is similar to that of the body and appendages (figure 1*b–d*), i.e. as calcite-filled voids, and they were presumably originally non-biomineralized.

A linear structure, oval in cross-section (long axis dorsoventrally), is preserved running subcentrally through the anterior part of the body, comprising 30–50% of body depth (figure 1*c*,*d*,*g,i,k*). Based on its appearance, position and size, this internal structure is interpreted as probably representing a portion of the gut, at least in part. Short, thin tabular features extend dorsolaterally and laterally from the area around the presumed gut towards the papillae (figure 1*c*,*d*,*g,i,k*). Additionally, narrow features extend ventrally for a short distance towards the axis of the lobopods (figure 1*d*,*g,i,k*). These three internal components continue posteriorly beyond their reconstructed length, but they become difficult to delimit and do not show any additional features.

## 4. Discussion

### 4.1. Systematics

Our phylogenetic analysis retrieved *T. distos* together with all three known *Hallucigenia* species as a clade in the panarthropod stem, sister to crown-group Panarthropoda (figure 2; electronic supplementary material, figure S1). Members of crown-group Panarthropoda are characterized by differentiated protocerebral appendages, which are lacking in the hallucigeniids and other stem-groups.

Caron & Aria [8] previously recovered *Cardiodictyon* [28] and *Carbotubulus* [16] with *Hallucigenia* (all three, known species) in a stem panarthropod clade. They expanded the concept of Hallucigeniidae, type genus *Hallucigenia* [26], to include these genera. The morphology of *T. distos* falls in general within their diagnosis, although it is distinguished by its tufted body papillae, and this difference together with others characteristic of the more restricted hallucigeniid clade recovered in our analysis are reflected in our revised family diagnosis. In our analysis, *Cardiodictyon* fell outside but immediately stemward of the clade including *T. distos* and Hallucigeniidae. We included *Carbotubulus* in initial runs of our analysis, but subsequently omitted it as the absence of many characters (particularly of its dorsal morphology) led to tree collapse. We found no support for the inclusion of *Microdictyon* in an expanded hallucigeniid clade (contra [8]).

Our hallucigeniid clade is characterized by a notable reduction in the length of the post-appendicular region, which is less than half the distance between the last two appendage pairs in *T*. *distos* and *Hallucigenia fortis*, and absent in *Hallucigenia sparsa* [29]. Seven or eight pairs of posterior (trunk) appendages are less slender than the anterior appendages, and bear terminal claws or a single claw. The number of trunk appendages excludes *Cardiodictyon* (some 25 pairs) and *Carbotubulus* (nine pairs) from the clade, restricting family Hallucigeniidae to just two genera, *Hallucigenia* and *Thanahita*. Caron & Aria [8] also recovered hallucigeniids stemward of crown-group panarthropods. The differences between our phylogenetic result and theirs reflect divergences both in analysis methodology (Bayesian in their case, parsimony in ours), and in the taxa- and character-sets. In particular, their matrix comprised only discrete characters, whereas ours incorporates continuous characters, as attempts to code our data as discrete resulted in low resolution in all parts of the tree.

Although details of the phylogeny vary depending on the taxa and characters included and methods used, there is a general consensus that lobopodians are paraphyletic (e.g. [4,7,8]). A more crownward placement of hallucigeniids, within the onychophoran stem-lineage, has been recovered by some studies [6,7]. This position implies that the shared architecture of the sclerotized elements of hallucigeniids [5], which retain previous sclerotized elements during growth resulting in a cone-in-cone structure, is an apomorphy of the clade. However, this feature is difficult to determine for most lobopodian taxa and its distribution is unclear; our analysis suggests, rather, that it is a plesiomorphic feature of Panarthropoda.

The other possible Silurian lobopodians, from North America, lack the tufted papillae of *T*. *distos* [13,15]. Additionally, the Waukesha species has 11 pairs of very short, proximally very broad, segmented trunk appendages and additional limbs in the head region, and that from Eramosa is similar.

### 4.2. Preservation and morphology

The *T*. *distos* holotype is fully three-dimensional and preserves traces of the presumed gut; like other Herefordshire fossils, it represents a carcass rather than a moult (e.g. [30–32]). It is clear that the entombing sediment rapidly became cohesive following burial, retaining a faithful external mould of the lobopodian [33]. In onychophorans, the body limb pairs show a strong tendency to move in phase with one another, and they exhibit metachronal rhythm, though they may exactly alternate or show any intermediate condition [34]. Thus, the relative position of the limbs on the specimen of *T*. *distos* (see description) does not correspond to any obvious onychophoran-related gait, and presumably reflects the nature of the burial event.

Fully three-dimensionally preserved Palaeozoic lobopodians are very rare. The only other examples are the two single, incomplete, phosphatized specimens of *Tritonychus phanerosarkus* and *Orstenotubulus evamuellerae* from, respectively, the lower Cambrian (Series 2, Stage 3) of Yunnan and the uppermost middle Cambrian (Series 3, Guzhangian Stage) Orsten of Sweden [35,36], and the single specimen of the smooth-surfaced *Carbotubulus waloszeki* from the Carboniferous Mazon Creek biota [16]. Three-dimensionally preserved fossil onychophorans are represented by the four incomplete specimens of *Cretoperipatus burmiticus* from the Cretaceous of Myanmar ([18,37]; but see also [19]). The style of preservation of *T. distos*—calcite void fill [33]—is different from that of the possible Silurian lobopodians from Waukasha and Eramosa, which are preserved in a combination of authigenic phosphatization and carbonaceous material [13–15]. They also occur in shallower water settings.

The papillae of *T*. *distos* contrast with the biomineralized sclerites of many other lobopodians, such as *Microdictyon* or *Cardiodictyon* (e.g. [27]). Their general form differs from the spinose projections present in many Cambrian species, such as in *Hallucigenia*, *Luolishania* or *Onychodictyon*, and invites comparison with the dermal papillae of some extant onychophorans, such as, for example, in *Eoperipatus* and *Epiperipatus* species ([38], fig. 10*e,f*), although those papillae are smaller (only a few hundreds of micrometres across) and more abundant. However, closely similar dermal papillae to those of onychophorans are also known, in *Tritonychus* and *Orstenotubulus* [35,36], from as early as Cambrian times.

The internal structures of the *T*. *distos* specimen are difficult to interpret. While their position is suggestive of a digestive tract, the tabular extensions towards the papillae (figure 1*c*,*d*,*g*,*i,k*,) may represent neural pathways. In extant onychophorans, there is a sensory bristle at the apex of some dermal papillae (cf. [38], fig. 10*b*). Also, it has been suggested that the spines of *Hallucigenia* and certain other lobopodians housed sensory (or secretory) structures [39], although we do not suggest that there is clear homology between spines and papillae. Similarly, comparison with living onychophorans suggests that the ventral extensions of internal structures towards appendages in *T*. *distos* (figure 1*g*,*i*,*k*) may represent the bases of limb nerves. However, neither the paired nature of the limb nerves nor the paired ventral nerve cord characteristic of extant species is evident in *T*. *distos* (figure 1*k*) [40, figs. 3*a*, 4*e*, 8]. Decay experiments on the onychophoran *Euperipatoides rowelli* showed that the gut was lost relatively rapidly but that it, and nervous tissue, survived longer than the body wall musculature [41]. We suggest that the internal structures preserved in *T*. *distos* represent a conflation of more than one organ system, including the digestive tract and at least part of the central nervous system.

Haug *et al*. [16] highlighted the difference in limb length in lobopodians. Taxa such as *Antennacanthopodia* from the early Cambrian (Series 2, Stage 3) Chengjiang Lagerstätte [42] bear stubby, conical short lobopods like those of extant onychophorans. By contrast, *Hallucigenia* from the lower and middle Cambrian (Series 2, Stage 3; Series 3, Stage 5), for example, and purportedly *Orstenotubulus* from the uppermost middle Cambrian (e.g. [5,27,36]), have slimmer, longer-legged lobopods. We could not place the longer-legged late Carboniferous *Carbotubulus* [16] in our phylogeny, and other long-legged forms (*Cardiodictyon* and *Microdictyon*) fall stemward of the hallucigeniid clade including *T*. *distos* (trunk limb ratio of 7 : 1 or 8 : 1) (figure 2). However, *T. distos* is the only long-legged post-Cambrian lobopodian known apart from *Carbotubulus* and this Silurian member of the hallucigeniid clade echoes reports of other soft-bodied taxa typical of the Cambrian in other Palaeozoic Lagerstätten (e.g. [10,43]).

## Supplementary Material

ESM Text S1

## Supplementary Material

ESM Figure S1

## Supplementary Material

ESM Table S1

## Supplementary Material

ESM Table S2

## Data accessibility

Serial grinding datasets and a virtual model of the *T*. *distos* holotype specimen in VAXML/STL format is available from the Dryad Digital Repository: http://dx.doi.org/10.5061/dryad.gk1g3 [44].

The character matrix dataset that forms the basis of the phylogenetic analysis is included in the electronic supplementary material (tables S1, S2).
