# Supplementary material for: A three-dimensionally preserved lobopodian from the Herefordshire (Silurian) Lagerstätte, UK
Source: R Soc Open Sci. 2018 Aug 8;5(8):172101. doi: 10.1098/rsos.172101 (PMC6124121; doi:10.1098/rsos.172101)
Supplement: ESM Text S1 [file rsos172101supp1.docx]

A three-dimensionally preserved lobopodian from the Herefordshire (Silurian) Lagerstätte, UK

Siveter Derek J., Briggs Derek E.G., Siveter David J., Sutton Mark D. & Legg, David

Electronic Supplementary Material text S1

1. METHODOLOGY

Details of the dataset and program used for phylogenetic analysis, together with relevant references, can be found in the main text under section (2.), Materials and Methods.

2. TAXA

Taxa were selected to aid comparison with other phylogenies of lobopodians (e.g. Ma *et al*. 2009). This requires being as comprehensive as possible in terms of taxon inclusion; however, several species were excluded, either *a priori* or *posteriori*. Earlier version of the dataset included: *Facivermis yunnanicus* Hou & Chen, 1989 (coded according to Liu *et al*. 2006a), *Tritonychus phanerosarkus* Zhang *et al*. 2016, *Helenodora inopinata* Thompson & Jones, 1980 (coded according to Murdock *et al*. 2016), and *Antennipatus montceauensis* Garwood *et al*. 2016. All were removed after a reduced consensus revealed them to be wildcard taxa, presumably due to a lack of preserved data. *Jianshanopodia decora* Liu *et al*. 2006b, is herein treated as a junior synonym of *Megadictyon haikouensis* Luo & Hu, *in* Luo *et al*. 1999, as unique apomorphies of these taxa could not be unequivocally distinguished.

Although *Carbotubulus waloszeki* Haug *et al*. 2012, has previously been allied to the hallucigeniids (Caron & Aria, 2017), the single, ventrally preserved specimen provides few reliable characters for coding. In an earlier version of the current data set this taxon was included but its position could not be adequately optimized. Until further specimens become available, particularly showing the dorsal morphology, then its position must remain open to question.

3. CHARACTERS

Characters were generally derived from previous studies of lobopodian relationships, particularly Smith & Ortega-Hernandez (2014), which has been used for several subsequent studies (e.g. Smith & Caron, 2015; Zhang *et al*. 2016), however, in some instances characters were modified from discrete to continuous characters. In some cases the methods of Randle and Sansom (2017) were used to aid selection of characters, such as spine length compared to spine width, or spine length compared to body width, by selecting these *a priori* and testing for independence. This was done to determine if these characters showed variation that was potentially significant for phylogeny rather than simply being a function of their construction.

3.1. Continuous

Continuous characters have been under-utilised in the phylogeny of panarthropods, but they are used herein as they serve in capturing more nuanced variation in closely related taxa and thus better elucidate potential relationships. Previous approaches have tended to treat variations in proportions as discrete characters and picked arbitrary “bins” as separate character states. Even if the distribution of measurements is bimodal, however, the spread of the data means that certain end member values are likely to be closer in value to that of another “bin” than those within its character group. This method is also likely to down-weight the importance of these characters in terms of evolutionary trend. For instance, if a lineage shows a directional evolution towards an increase in length of a particular

element, such as the antenna, this trend will not be observed with discrete characters. Many classic studies of evolution demonstrate the evolution of characters as shifts in a normalised bell-shaped curve, whereby end members show a selective advantage causing the bell curve to shift in subsequent generations. In this way most, if not all characters, can be thought of as a gradual shift in proportions over time and show some form of gradational evolution. Continuous characters are therefore more likely to capture the actual mode of evolution than discrete characters, and for this reason they were also used in the current study to capture aspects of shape, rather than using discrete end members.

When devising continuous characters, it is important to consider character independence, as their production often involves comparison with other elements. Such comparisons are also made when devising discrete characters. It is almost impossible for a character to be entirely independent of another. However, in order to ensure that certain measurements were independent of each other, an independence test employed by Randall & Sansom (2017) was utilised. In our study denominators were selected because they were instrumental in determining whether variation was actually visible in a character. For instance, we often used trunk dimensions as they provide a useful, practical comparator for determining whether elements attached to the trunk are considered long or short, e.g. trunk spines. Using the trunk had no undue effect on character variation when tested for independence, thus ensuring it did not add any undue weighting to the character or over-emphasise the importance of trunk width as a variable.

Inapplicable characters for continuous character states are coded as “?” rather than “-“, however, to distinguish inapplicable character states from missing data in the online supplementary matrix they are coloured differently, with inapplicable character states represented with a darker shade than missing data.

For previously described species, measurements were made from published photos using ImageJ. When measurements were made from repeated body elements, e.g. a series of lobopodous limbs, and/or from numerous specimens, values are given as ranges. All values, usually expressed as a decimalized ratio, are given to two decimal places. Widths were selected as denominators where possible for consistency.

3.1.1. Anterior sclerite

0. *Length to width ratio of medial anterior sclerite*.

This character is inapplicable for species lacking an anterior sclerite (character **39**). Measurement was taken exclusively from dorso-ventrally preserved specimens, measured along their sagittal axis, and transversely at the widest point of the anterior sclerite. The former was then divided by the latter. Variation in anterior sclerite dimensions have previously been used to distinguish between closely related species of hurdiid (Daley *et al*. 2013). The latter, including *Aegirocassis benmoulae*, *Hurdia victoria*, and *Peytoia nathorsti*, tend to possess an anterior sclerite that is longer than wide, with values ranging from 2.45 in *A. benmoulae* (YPM 227556; Van Roy *et al*. 2015, Extended Data Fig. 3c), to 1.15 in *P. nathorsti* (USNM 274143; Whittington & Briggs, 1985, Fig. 17), whilst anomalocaridids and upper stem-group euarthropods possess one that is wider than long, ranging from 0.84 in *Anomalocaris canadensis* (ROM 51212; Daley & Edgecombe, 2014, Fig. 4.1), to 0.40 in *Fuxianhuia protensa* (CN 115354; Hou & Bergström, 1997, Fig. 9C).

3.1.2. Trunk

1. *Length of dorso-lateral body spines, compared to total body width.*

This character is inapplicable for species lacking dorso-lateral body spines (character **60**). Measurements were mostly taken from laterally preserved specimens, measured along the anterior margin of the body spines, and dorso-ventrally along the widest part of the lopododous trunk. This character serves to distinguish the short dorso-lateral spines of some luolishaniids and *Onychodictyon* spp. from the relatively long spines of hallucigeniids, which can measure over 6x body width (e.g. *Hallucigenia sparsa*, ROM 63139, Smith & Caron, 2015, Extended Data Fig. 2a).

2. *Length to width ratio of dorso-lateral body spines*.

This character is inapplicable for species lacking dorso-lateral body spines (character **60**). Measurement is taken along the anterior length of the spine, and across the spine base.

3. *Length of post-appendicular extension, compared to average inter-appendicular length*.

This character is inapplicable for species lacking a post-appendicular extension (character **65**).

3.1.3. Appendages

*Protocerebral (pre-ocular)*

4. *Length of protocerebral appendage(s), compared to total cephalic width*.

This character is inapplicable for species lacking differentiated protocerebral appendages (character **73**).

5.  *Length of primary spine(s), compared to main body of parent podomere*.

This character is inapplicable for species lacking spinose projections on their protocerebral appendages (character **81**).

*Post-tritocerebral (Trunk)*

*Exites*.

6. *Relative difference in length between anterior-most body exite(s) (excluding “neck” or hypertrophied exites), and posterior-most body exite(s) (excluding lateral processes)*.

This character is inapplicable for taxa lacking distinct exites (character **94**).

*Endopod.*

7. *Length to width ratio of inner appendicular branch (endopod or equivalent)*.

This character, and others in this section, are inapplicable for taxa lacking an inner limb-branch or equivalent (character **98**). This character serves to distinguish the elongate appendages of most lobopodians and upper-stem-group arthropods, from the short appendages of onychophorans and the flap-like inner rami of dinocaridids (Van Roy *et al*. 2015).

8. *Length of anterior lobopodous body appendages, compared to total body width*.

This character is only applicable to lobopodians showing a clear pseudotagmotic distinction, usually in terms of length and ornamentation, between their anterior and posterior appendage pairs, specifically hallucigeniids and luolishaniids.

9. *Length of posterior lobopodous body appendages, compared to total body width*.

This character applies to all taxa bearing lobopodous appendages, including those lacking distinct pseudotagmatic division of the appendages (see character 51). In the latter case, an average is taken of the posterior three appendage pair lengths.

10. *Average length of anterior lobopodous body appendages, compared to posterior lobopodous body appendage*.

This character is only applicable to those taxa bearing two distinct pseudotagmatic divisions of the lobopodous appendages (see character 51).

3.2. Meristic

Meristic (countable) characters have generally been treated in a discrete manner, however, to increase information content and better identify trend these characters are analysed as continuous characters herein.

3.2.1. Anterior sclerite

11. *Number of dorsal protocerebral sclerites*.

This character is inapplicable for taxa lacking a distinct sclerite in their anterior cephalic region (character **39**). The majority of taxa possess a single cephalic sclerite, however, the luolishanids *Collinsium cilliosum* Yang *et al*. 2015, and the currently undescribed ROM 52703 and 53234 (DAL pers. ob.), possess at least two, whilst *Loulishania* has three.

3.2.2. External ocular features

12. *Number of lateral eye pairs*.

3.2.3. Trunk

13. *Number of raised nodes (including spines and sclerites) per segment*.

14. *Number of arthrodized trunk segments (excluding telson)*.

3.2.4. Appendages

15. *Number of head appendage pairs*.

16. *Number of body appendage pairs*.

17. *Number of appendage pairs in anterior pseudotagma*.

This character is only applicable to taxa possessing a body that is divided into two pseudotagmata, distinguished by the morphology of their lobopodous appendages (see character 51).

18. *Number of setal rows in anterior body appendage*.

19. *Number of terminal claws, per appendage, in the anterior pseudotagma*.

20. *Number of appendage pairs in the posterior pseudotagma*.

This character is only applicable to taxa possessing a body that is divided into two pseudotagmata, distinguished by the morphology of their lobopodous appendages (see character 51).

21. *Number of terminal claws, per body appendage (excluding anterior pseudotagmata)*.

*Protocerebral (pre-ocular)*

22. *Number of coplanar protocerebral spine rows*.

23. *Number of podomeres in each protocerebral appendage*.

*Deutocerebral*

24. *Number of post-protocerebral cephalic appendage pairs*.

*Tritocerebral*

25. *Number of podomeres in each tritocerebral appendage*.

*Post-tritocerebral (Trunk)*

*Exites*.

26. *Number of segments in each exite*.

27. *Number of posterior exite pairs modified into lateral telson processes*.

*Endopods.*

28. *Number of podomeres in each trunk appendage*.

3.2.5. Neurological

29. *Number of constituent neuromeres in dorsally condensed brain*.

[See Smith & Ortega-Hernandez (2015) for a justification of coding]

3.3. Discrete

3.3.1. Integument

30. *Metameric segmentation of the antero-posterior body axis: (0) absent, (1) present*.

The definition of a segment has undergone numerous iterations (Hannibal & Patel, 2013). Here a segment is defined in the loosest sense as the external expression of a repeated body unit and thus includes both *eusegments* and *pseudosegments* (*sensu* Minelli & Fusco, 2004). This is most obvious in the sclerotized trunk integument of euarthropods, which consists of serially repeated, limb-bearing somites, and the repeated limb pairs of onychophorans and tardigrades. Segmentation, in this sense, is lacking in the cycloneuralians *Caenorhabditis elegans* and *Priapulus caudatus*.

31. *Epicuticular pillars: (0) absent, (1) present*.

The inner epicuticular layer of many tardigrades possess elongate, pillar-like structures with a circular cross-section and wide extremities (Greven, 1984). Epicuticular pillars have been reported from a single specimen of a middle Cambrian tardigrade, UB W 266 (Müller *et al*., 1995), the heterotardigrade *Echiniscus testudo* (Greven, 1971), and from the arthrotardigrades *Stygarctus bradypus* (Hansen *et al*., 2012), and *Actinarctus doryphorus* (Shulze *et al*., 2014), which has led some to consider this feature a syanpomorphy of total group Tardigrada (Maas & Waloszek, 2001). They are, however, absent from the majority of tardigrades included in the current analysis, including *Macrobiotus hufelandi* (Baccetti & Rosati, 1971), *Milnesium tardigradum* (Greven & Robenek, 1983), and *Echiniscoides sigismundi* (Greven & Grohé, 1975). Epicuticular pillars were reported from the hypsibiid *Isohypsibius sculptus* (Dastych, 1997), although they are apparently absent from all other hypsibiids, specifically the one included in the current analysis, *Hypsibius dujardini*.

Both outgroups, namely *Caenorhabditis elegans* and *Priapulus caudatus*, possess a membrane-like epicuticle lacking internal structures (Wright, 1987; Lemburg, 1998), and are thus coded as absent for this character. Specific details regarding the epicuticle of onychophorans used in this analysis could not be found and thus this character is coded as uncertain for all exemplars included herein.

32. *Sclerotized elements (including body nodes and claw) composed of stacks of constituent elements: (0) absent, (1) present*.

33. *Rigid (sclerotized) body cuticle: (0) absent, (1) present*.

34. *Cuticle arthrodized: (0) absent, (1) present*.

3.3.2. Anterior tagmata

35. *Distinct external cephalization: (0) absent, (1) present*.

Cephalization is loosely defined as the concentration of sensory organs systems, both internal and external, often associated with a preferred direction of motion, at the anterior of the main body axis (Brusca & Brusca, 2002). Although of considerable importance in the evolution of various bilaterian phyla (Cavalier-Smith, 2017), such a broad definition makes it incredibly hard to code as a character and is potentially phylogenetically uninformative, particularly with regards to fossils, in which internal features are often lacking and function is hard, if not impossible, to infer. For this reason, the external expression of cephalization, in the form of a differentiated anterior body terminus, is coded instead. This differentiation may take the form of a change in dimensions with regard to the trunk, such as the bulbous anterior region of *Hallucigenia fortis* (Ramsköld & Chen, 1998), or the possession of potential sensory organs, such as eyes or differentiated appendages. In this regard, this character resembles character 25 of Ma *et al*. (2009) – “[d]istinct head”, and is coded in a similar manner. An externally differentiated anterior body terminus is lacking in *Paucipodia inermis*, *Diania cactiformis* (*contra* Liu *et al*., 2011), and the outgroup *Caenorhabditis elegans*. Although *Priapulus caudatus* has a differentiated anterior region, in the form of an introvert, this is considered part of an extensive oral architecture and not part of a distinct cephalic region as described herein, and thus this character is scored as absent for this particular taxon. Also, although often lacking external sensory organs the anterior body terminus of tardigrades shows a notable differentiation from the posterior segments and they are thus coded as possessing a distinct external cephalization.

36. *Cephalic papillae: (0) absent, (1) present*.

37. *Medial cephalic cirri: (0) absent, (1) present*.

38. *Lateral cephalic cirri: (0) absent, (1) present*.

3.3.3. Anterior sclerite (and external ocular features)

39. *Rigid (sclerotized) dorsal covering of the protocerebral somite: (0) absent, (1) present*.

An anterior dorsal cephalic covering, associated with the protocerebral somite, is common amongst panarthropods, appearing in tardigrades, luolishaniids, dinocaridids, and upper stem-group euarthropods (*sensu* Budd, 2008). In tardigrades, this covers the entire prosomal somite and is called the *cephalic plate*. It is present in *Stygarctus bradypus* (Hansen *et al*., 2012), and *Echiniscus testudo* (Greven, 1984), but lacking in all other tardigrades present in the current analysis. The posterior of the presumed prosomal somite of some luolishaniids possesses one, two, or three, hardened cuticular plates. A single rounded sclerite was reported for SAM P 14848 (García-Bellido *et al*. 2013), whilst three, two lateral and one medial, conical sclerites are present in *Luolishania longicruris* (Ma *et al*., 2009), and two similar dorsolateral spines in *Collinsium ciliosum* (Yang *et al*., 2015), and ROM 53234 (DL pers. obs.). This structure appears to be absent in ROM 52703 (DL pers. obs.), although preservation is too poor in the cephalic region to be sure, and thus this character is scored as uncertain for this taxon. Amongst other lobopodians, a scleritized cephalic covering has also been reported for *Hallucigenia fortis*, *Cardiodictyon catenulum* (both in Hou & Bergström, 1995), and *Onychodictyon ferox* (Ramsköld & Chen, 1998; Ou *et al*., 2012), although all have since been shown to lack this feature (Ramsköld & Chen, 1998; Liu & Dunlop, 2014).

The anterior cephalic plate of dinocaridids and upper stem-group euarthropods is termed the *ocular* or *anterior sclerite* (*sensu* Budd, 2008), and as the name suggests is usually associated with a pair of lateral eyes. In dinocaridids this can be subtriangular, and quite large, covering the entirety of the cephalic region, such as in *Aegirocaris benmoulae* (Van Roy *et al*. 2015), or slightly smaller and rounded, as in *Anomalocaris canadensis* (Daley & Edgecombe, 2014). In the hurdiids, namely *A. benmoulae*, and *Hurdia victoria*, the anterior sclerite, or *H-element* (*sensu* Daley *et al*., 2009), is associated with two additional lateral plates, termed *P-elements* (*sensu* Daley *et al*., 2009). The homology of these elements to structures in other arthropods, specifically the carapace of upper stem-group euarthropods, has been suggested (Legg & Vannier, 2013), however, their association with the anterior sclerite indicates they originate from the protocerebral somite.

The anterior sclerite of upper stem-group euarthropods is limited to the ocular segment (Budd, 2008), and often disassociated from the posterior sclerotized cephalic elements. Presumable in other arthropods this structure has become fused to the cephalic shield, however, because this cannot be demonstrated with certainty, this character is coded as absent for taxa lacking a distinct, subovoid anterior sclerite. Amongst taxa present in the current analysis, such a structure can be observed in *Nereocaris briggsi* (Legg & Caron, 2014), *Fuxianhuia protensa* (Bergström *et al*., 2008), and *Chengjiangocaris kunmingensis* (Yang *et al*. 2013).

40. *Distribution of anterior (protocerebral) somite: (0) covering the majority of the cephalic region, (1) limited to anterior cephalic region (or eye stalk)*.

41. *Anterior tapering of anterior sclerite: (0) absent, (1) present*.

42. *Medial carina on anterior sclerite: (0) absent, (1) present*.

43. *Marginal rim on anterior sclerite: (0) absent, (1) present*.

3.3.4. External ocular features

44. *Medial eye: (0) absent, (1) present*.

45. *Lateral eyes: (0) absent, (1) present*.

46. *Lateral eyes multi-facetted: (0) absent, (1) present*.

47. *Lateral eye facets concentrated into compound eyes: (0) absent, (1) present*.

48. *Eye stalks: (0) absent (eyes sessile), (1) present*.

3.3.5. Cephalon

49. *Rigid (sclerotized) syntergite covering multiple dorsal anterior somites: (0) absent, (1) present*.

3.3.6. Carapace

50. *Posterior and lateral tergal extensions of the cephalic region: (0) absent, (1) present*.

3.3.7. Body

51. *Body divided into two pseudotagmata: (0) absent, (1) present*.

This character is only applicable to taxa possessing an unsclerotized body (character **34**). The reason for this is because it is unclear if the pseudotagmata of lobopodians, correspond in any way to the pseudotagmata of sclerotized taxa, mostly because the criteria for identifying these units in the former, such as variations in spine and claw morphology, are not present in the latter. Pseudotagmata in this case are defined as the specialization of different body region, as expressed in their limbs, or variation in the length of trunk spines. Both, hallucigeniids and luolishanids demonstrate a form of pseudotagmatization. In the former, the anterior most appendages are shorter, and slimmer than the posterior appendages and lack terminal claws, a condition also observed in *Cardiodictyon* (Liu and Dunlop, 2014), whilst in luolishanids the anterior appendages are elongate, slim, possess numerous terminal claws, and are fringed with setae.

52. *Prominent trunk annulation: (0) absent, (1) present*.

Although annulation has been observed in a large number of lobopodian taxa, this character only applies for those taxa in which annulation is a permanent feature, i.e. rather than the result of wrinkling during cuticular flexing.

53. *Body papillae: (0) absent, (1) present*.

54. *Papillae with squamose ornamentation: (0) absent, (1) present*.

The most prominent papillae of onychophorans are covered by a coating of smaller papillae, giving a squamose appearance.

55. *Divided trunk papillae: (0) absent, (1) present*.

The papillae of peripatids, specifically *Peripatus juliformis*, *Euperipatus edwardsii*, and the Cretaceous *Cretaperipatus burmiticus*, are divided into two prominent regions.

56. *Elevated body nodes: (0) absent, (1) present*.

57. *Sclerotization of body nodes: (0) absent, (1) present*.

58. *Elevated body nodes (including spines and sclerites) situated above anterior body appendages: (0) absent, (1) present*.

59. *Ornamentation of sclerotized body nodes: (0) porous, (1) squamose*.

60. *Body nodes extended into spines: (0) absent, (1) present*.

61. *Each body spine situated on a raised, unsclerotized base: (0) absent, (1) present*.

62. *Medial spine: (0) absent, (1) present*.

63. *Bases of spines fused into a sclerotized body ring: (0) absent, (1) present*.

64. *Dorsal band of lanceolate setae: (0) absent, (1) present*.

65. *Post-appendicular extension: (0) absent, (1) present*.

3.3.8. Trunk

66. *Arthrodized cuticle extended into distinct dorsal tergites: (0) absent, (1) present*.

67. *Sternites: (0) absent, (1) present*.

68. *Trunk divided into two distinct pseudotagmata, an anterior thorax, and posterior abdomen: (0) absent, (1) present*.

This character refers to the specialization of dorsal segments into distinct pseudotagmata as demonstrated by a difference in tergite morphology and is thus inapplicable for taxa lacking distinct dorsal tergites (character **66**).

69. *Pygidium: (0) absent, (1) present*.

70. *Telson: (0) absent, (1) present*.

71. *Telson bearing a setal fringe: (0) absent, (1) present*.

3.3.9. Appendages

72. *Paired ventro-lateral appendages: (0) absent, (1) present*.

*Protocerebral (pre-ocular)*

73. *Differentiated protocerebral appendages: (0) absent, (1) present*.

This character refers to the anteriormost pair of appendages in many lobopodians, the frontal appendages of dinocaridids, and the hypostome of deuteropods, which are all considered to derive from the protocerebral somite (see discussion in Ortega-Hernández & Budd, 2016), and are different in morphology and/or orientation from the more posterior limbs. Although it is possible that the anteriormost limbs of certain lobopodians also belong to the protocerebral somite, they are not differentiated from more posterior limbs. For instance, hallucigenids show a series of limbs in the anterior but the anteriormost of these is not differentiated from the remaining pairs in this series and is therefore coded as absent for this character. Also, the great distance between the putative ocular elements and the first pair of appendages also makes it unlikely they both originate from the same ganglion, which is presumed to be protocerebral for the eyes based on comparisons with other panarthropods. In *Antennacanthopodia* the first pair of appendages is associated with a pair of putative eyes and they are differentiated from the remaining trunk segments; this pair is therefore coded as present for this character. The second pair of appendages, albeit similar in morphology to the preceding (protocerebral) appendage, are considered a differentiated pair of deutocerebral appendages, and are thus distinct from the trunk appendages.

74. *Location of protocerebral appendages: (0) internal, (1) external*.

This character serves to distinguish the internal protocerebral appendages of tardigrades, termed the stylet apparatus, from the more appendicular external primary antennae and grasping appendages or lobopodians and dinocaridids.

75. *Orientation of external protocerebral appendage: (0) dorsal, (1) lateral, (2) ventral*. [ADDITIVE]

76. *Protocerebral appendages with prominent, heteronomous annulation: (0) absent, (1) present*.

77. *Rigid (sclerotized) protocerebral appendages: (0) absent, (1) present*.

78. *Arthropodization of rigid (sclerotized) protocerebral appendages: (0) absent, (1) present*.

79. *Medial fusion of protocerebral appendages: (0) absent, (1) present*.

80. *Extent of appendicular fusion: (0) basal, (1) entire*.

81. *Spinose projections on protocerebral appendages: (0) absent, (1) present*.

82. *Orientation of primary spine row: (0) dorsal, (1) mediolateral, (1) ventral*.

83. *Primary spines bearing secondary spines: (0) absent, (1) present*.

84. *Orientation of auxiliary spines: (0) terminal, (1) lateral*.

85. *Coplanar auxiliary spines, oppositional to primary spine row: (0) absent, (1) present*.

86. *Orientation of terminal spine(s) in relation to primary spine row: (0) in same orientation, (1) in oppositional orientation*.

87. *Multifurcate distal termination on protocerebral appendage(s): (0) absent, (1) present*.

*Deutocerebral*

88. *Differentiated deutocerebral appendages: (0) absent, (1) present*.

89. *Nature of differential deutocerebral appendages: (0) appendicular, (1) sclerotized jaws*.

90. *Diastema on sclerotized jaws: (0) absent, (1) present*.

*Tritocerebral*

91. *Geniculate tritocerebral appendages: (0) absent, (1) present*.

*Post-tritocerebral (Trunk)*

92. *Fusion of endopods and exites into biramous appendages: (0) absent, (1) present*.

*Protopodites.*

93. *Distinct protopodite: (0) absent, (1) present*.

*Exites*.

94. *Exites: (0) absent, (1) present*.

95. *Transverse lineations on exites: (0) absent, (1) present*.

96. *Exites fringes with setae: (0) absent, (1) present*.

97. *Setae on exites imbricated: (0) absent, (1) present*.

*Endopod (or equivalent).*

98. *Endopods (or equivalent): (0) absent, (1) present*.

99. *Prominent heteronomous appendicular annulation: (0) absent, (1) present*.

100. *Telescoping of appendicular annuli: (0) absent, (1) present*.

101. *Post-tritocerebral appendages sclerotized and arthropodized: (0) absent, (1) present*.

102. *Setae on lobopodous appendages.*

103. *Endites on arthropodized endopods: (0) absent, (1) present*

104. *Terminal pads: (0) absent, (1) present*.

105. *Digitate distal extensions of the appendage: (0) absent, (1) present*.

106. *Appendages bearing terminal claw(s): (0) absent, (1) present*.

107. *Anterior terminal claw(s) differentiated from posterior terminal claw(s) on same appendage: (0) absent, (1) present*.

108. *Terminal claws with secondary spines or serration: (0) absent, (1) present*.

109. *Terminal claws on appendages in anterior pseudotagmata: (0) absent, (1) present*.

*Posterior appendages*

110. *Terminal appendages meet at postero-medial body axis: (0) absent, (1) present*.

111. *Serration limited to claws on terminal appendage pair: (0) absent, (1) present*.

112. *Orientation of posterior-most terminal claws different from anterior claws: (0) absent, (1) present*.

3.3.10. Digestive

113. *Orientation of mouth: (0) terminal, (1) ventral, (2) posterior*. [ADDITIVE]

114. *Radially arranged circumoral structure: (0) absent, (1) present*.

115, *Nature of radially arranged circumoral structure: (0) scalids, (1) papillae, (2) lamellae, (3) sclerites*.

116. *Overlap of oral sclerites: (0) absent, (1) present*.

117. *Differentiated oral sclerites: (0) absent, (1) present*.

118. *Symmetrical arrangement of oral sclerites: (0) absent, (1) present*.

119. *Squamose ornamentation of oral sclerites: (0) absent, (1) present*.

120. *Proboscis: (0) absent, (1) present*.

121. *Lateral gut glands: (0) absent, (1) present*.

3.3.11. Neurological

122. *Dorsal condensed brain: (0) absent, (1) present*.

123. *Paired ventral nerve cord: (0) absent, (1) present*.

124. *Ventral nerve cord ganglionated: (0) absent, (1) present*.

125. *Ventral nerve cord lateralized: (0) absent, (1) present*.

3.3.12. Reproductive

126. *Ovoviviparity: (0) absent, (1) present*.

127. *Placenta: (0) absent, (1) present*.

4. EXTENDED RESULTS

Analysis using the method outline above produced a single most parsimonious tree of 22.34123 steps (Supplementary Figure 1). *Thanahita distos* gen. et sp. nov. resolved as part of a polytomy containing *Hallucigenia hongmeia* and a clade composed of *Hallucigenia fortis* and *H. sparsa*. A reduced consensus revealed this lack of resolution to be due to the incompleteness of *H. hongmeia*, as numerous important characters which might better reveal its systematic position are not preserved in the available material. This clade, consisting of *H. hongmeia*, *H. fortis*, and *H. sparsa*, hereafter referred to as Hallucigeniidae, was supported by just two unequivocal synapomophies, (ch. 3) a reduction in post-appendicular extension length, and (ch. 20) the possession of seven differentiated appendages in a posterior pseudotagma.

The position of hallucigeniids amongst panarthropods has been contentious, with more recent papers (e.g. Smith & Ortega-Hernández, 2014) suggesting a close relationship with onychophorans based on the similarities in construction of sclerotized elements such as spines and jaws. Unfortunately, this particular feature could not be adequately optimized on the current phylogeny as the growth mode of the sclerotised elements of many lobopodian taxa is unknown and it is entirely possible based on our current knowledge that this feature represents a synplesiomophic feature of panarthropods rather than a genuine synapomophy of Onychophora+Hallucigeniidae. Other features present in the current analysis favour an alternative position for hallucigeniids outside of crown-group panarthropods, such as their lack of unequivocal, and differentiated protocerebral appendages, a feature otherwise shared between all unequivocal panarthropods.

**REFERENCES**

Baccetti, B. & Rosati, F. (1971). Electron microscopy on tardigrades. III. The integument. *Journal of Ultrastructure Research*, **34**:214—243.

Bergström, J., Hou, X., Zhang, X. & Clausen, S. (2008). A new view of the Cambrian arthropod *Fuxianhuia*. *GFF*, **130**:189—201.

Brusca, R.C. & Brusca, G.J. (2002). *Invertebrates. Second Edition.* Sinauer Associates, Ltd. 936 pp.

Budd, G.E. (2008). Head structure in upper stem-group euarthropods. *Palaeontology*, **51**:561—573.

Caron, J.-B. & Aria, C. (2017). Cambrian suspension-feeding lobopodians and the early radiation of panarthropods. *BMC Evolutionary Biology*, **17**:29.

Cavalier-Smith, T. (2017). Origin of animal multicellularity: precursors, causes, consequences—the choanoflagellate/sponge transition, neurogenesis and the Cambrian explosion. *Philosophical Transactions of the Royal Society B*, **372**:20150476.

Daley, A.C. & Edgecombe, G.D. (2014). Morphology of *Anomalocaris canadensis* from the Burgess Shale. *Journal of Paleontology*, **88**:69—91.

Daley, A.C., Budd, G.E., Caron, J.-B., Edgecombe, G.D. & Collins, D. (2009). The Burgess Shale anomalocaridid *Hurdia* and its significance for early euarthropod evolution. *Science*, **323**:1597—1600.

Daley, A.C., Budd, G.E. & Caron, J.-B. (2013). Morphology and systematics of the anomalocaridid arthropod *Hurdia* from the Middle Cambrian of British Columbia and Utah. *Journal of Systematic Palaeontology*, **11**:743—787.

Dastych, H. (1997). Redescription of *Isohypsibius sculptus* Ramazzotti, 1962 (Tardigrada). *Entomologische Mittelungen aus dem Zoologischen Museum Hamburg*, **12**:163—167.

García-Bellido, D.C., Edgecombe, G.D., Paterson, J.R. & Ma, X. (2013). A ‘Collins’ monster’-type lobopodian from the Emu Bay Shale Konservat-Lagerstátte (Cambrian), South Australia. *Alcheringa*, **37**:1—5.

Garwood, R.J., Edgecombe, G.D., Charbonnier, S., Chabard, D., Sotty, D. & Giribet, G. (2016). Carboniferous Onychophora from Montceau-les-Mines, France, and onychophoran terrestrialization. *Invertebrate Biology*, **135**:179—190.

Goloboff, P.A. (1993). Estimating character weights during tree search. *Cladistics*, **9**:83—91.

Goloboff, P.A., Farris, J.S., Källersjö, M., Oxelman, B. & Szumik, C.A. (2003). Improvements to resampling measures of group support. *Cladistics*, **19**:324—332.

Goloboff, P.A., Farris, J.S. & Nixon, K.C. (2008). TNT, a free program for phylogenetic analysis. *Cladistics*, **24**:1—13.

Greven, G. (1971). Zur Feinstruktur der inneren Epicuticula von *Eschiniscus testudo*. *Naturwissenschaften*, **58**:367—368.

Greven, H. (1984). Tardigrada. pp. 714—727. *In* Bereiter-Hahn, J., Matoltsy, A.G. & Richards, K.S. (eds.). *Biology of Integument: Invertebrates. Volume 1.* Springer-Verlag, New York.

Greven, H. & Grohé, G. (1975). Die Feinstruktur des Integumentes und der Muskelansatzstellen von *Echiniscoides sigismundi* (Heterotardigrada). *Helgoländer wissenschaftliche Meeresuntersuchungen*, **27**:450—460.

Greven, H. & Robenek, H. (1983). Freeze-fracture studies on tardigrade cuticle. *Tissue & Cell*, **15**:329—340.

Hannibal, R.L. & Patel, N.H. (2013). What is a segment? *EvoDevo*, **4**:35.

Hansen, J.G., Kristensen, R.M. & Jørgensen, A. (2012). *The armoured marine tardigrades (Arthrotardigrada, Tardigrada)*. Det Kongelige Danske Videnskabernes Selskab. 91 pp.

Haug, J.T., Mayer, G., Haug, C. & Briggs, D.E.G. (2012). A Carboniferous non-onychophoran lobopodian reveals long-term survival of a Cambrian morphotype. *Current Biology*, **22**:1673—1675.

Hou, X. & Bergström, J. (1995). Cambrian lobopodians-ancestors of extant onychophorans? *Zoological Journal of the Linnean Society*, **114**:3—19.

Hou, X. & Bergström, J. (1997). Arthropods of the Lower Cambrian Chengjiang fauna, southwest China. *Fossils & Strata*, **45**:1—116.

Hou X.-G. & Bergström, J. 2006: Dinocaridids: anomalous arthropods or ar- thropod-like worms? *In* J.-Y. Rong, Z.-J. Fang, Z.-H. Zhanghe, R.-B. Zhann, X.-D. Wang & X.-L. Yuan (eds.): *Originations, Radiations and Biodiversity Changes – evidences from the Chinese fossil record*, 139–158 (in Chinese), 847–850 (extended abstract in English). Science Press, Beijing.

Hou, X. & Chen, J. (1989). Early Cambrian tentacled worm-like animals (*Facivermis* gen. nov.) from Chengjiang, Yunnan. *Acta Palaeontologica Sinica*, **28**:32—41.

Legg, D.A. & Caron, J.-B. (2014). New Middle Cambrian bivalved arthropods from the Burgess Shale (British Columbia). *Palaeontology*, **57**:691—711.

Legg, D.A. & Vannier, J. (2013). The affinities of the cosmopolitan arthropod *Isoxys* and its implications for the origin of arthropods. *Lethaia*, **46**:540—550.

Lemburg, C. (1998). Electron microscopical localization of chitin in the cuticle of *Halicryptus spinulosus* and *Priapulus caudatus* (Priapulida) using gold-labelled wheat germ agglutinin: phylogenetic implications for the evolution of the cuticle within the Nemathelminthes. *Zoomorphology*, **118**:137—158.

Liu, J. & Dunlop, J.A. 2014. Cambrian lobopodians: A review of recent progress in our understanding of their morphology and evolution. *Palaeogeography*, *Palaeoclimatology*, *Palaeoecology*, **398**: 4-15.

Liu, J., Han, J., Simonetta, A.M., Hu, S., Zhang, Z., Yao, Y. & Shu, D. (2006a). New observations of the lobopod-like worm *Facivermis* from the Early Cambrian Chengjiang Lagerstätte. *Chinese Science Bulletin*, **51**:358—363.

Liu, J., Shu, D., Han, J., Zhang, Z. & Zhang, X. (2006b). A large xenusiid lobopod with complex appendages from the Lower Cambrian Chengjiang Lagerstätte. *Acta Palaeontologica Polonica*, **51**:215—222.

Liu, J., Steiner, M., Dunlop, J.A., Keupp, H., Shu, D., Ou, Q., Han, J. & Zhang, Z. (2011). An armoured Cambrian lobopodian from China with arthropod-like appendages. *Nature*, **470**:526—530.

Luo, H., Hu, S., Chen, L., Zhang, S. & Tao, Y. (1999). *Early Cambrian Chengjiang fauna from Kunming region China*. Yunnan Science and Technology Press, Kunming, China. 129 pp.

Ma, X., Hou, X. & Bergström, J. (2009). Morphology of *Luolishania longicruris* (Lower Cambrian, Chengjiang Lagerstätte, SW China) and the phylogenetic relationships within lobopodians. *Arthropod Structure & Development*, **38**:271—291.

Maas, A. & Waloszek, D. (2001). Cambrian derivatives of the early arthropod stem lineage, pentastomids, tardigrades and lobopodians – an ‘Orsten’ perspective. *Zoologischer Anzeiger*, **240**:451—459.

Minelli, A. & Fusco, G. (2004). Evo-devo perspectives on segmentation: model organisms, and beyond. *Trends in Ecology and Evolution*, **19**:423—429.

Müller, K.J., Walossek, D. & Zakharov, A. (1995). ‘Orsten’ type phosphatized soft-integument preservation and a new record from the Middle Cambrian Kuonamka Formation in Siberia. *Neues Jahrbuch für Geologie und Palaeontologie (Abh.)*, **191**:101—118.

Murdock, D.J.E., Gabbott, S.E. & Purnell, M.A. (2016). The impact of taphonomic data on phylogenetic resolution: *Helenodora inopinata* (Carboniferous, Mazon Creek Lagerstätte) and the onychophoran stem lineage. *BMC Evolutionary Biology*, **16**:19.

Ou, Q., Shu, D. & Mayer, G. (2012). Cambrian lobopodians and extant onychophorans provide new insights into early cephalization in Panarthropoda. *Nature Communications*, **3**:1261.

RANDLE, E. & SAMSON, R.S. (2017). Exploring phylogenetic relationships of Pteraspidiformes heterostracans (stem-gnathostomes) using continuous and discrete characters. *Journal of* *Systematic* *Palaeontology*, **15**, 583-599.

Ramsköld, L. & Chen, J. (1998). Cambrian lobopodians: morphology and phylogeny. pp. 107—150. *In* Edgecombe, G.D. (ed.). *Arthropod Fossils and Phylogeny*. Columbia University Press, New York.

Shulze, C., Neves, R.C. & Schmidt-Rhaesa, A. (2014). Comparative immunohistochemical investigation on the nervous system of two species of Arthrotardigrada (Heterotardigrada, Tardigrada). *Zoologischer Anzeiger*, **253**:225—235.

Smith, M.R. & Caron, J.-B. (2015). *Hallucigenia*’s head and the pharyngeal armature of early ecdysozoans. *Nature*, **523**:75—78.

Smith, M.R. & Ortega-Hernández, J. (2014). *Hallucigenia*’s onychophoran-like claws and the case for Tactopoda. *Nature*, **514**:363—366.

Thompson, I. & Jones, D.S. (1980). A possible onychophoran from the Middle Pennsylvanian Mazon Creek beds of Northern Illinois. *Journal of Paleontology*, **54**:588—596.

Van Roy, P., Daley, A.C. & Briggs, D.E.G. (2015). Anomalocaridid trunk limb homology revealed by a giant filter-feeder with paired flaps. *Nature*, **522**:77—80.

Whittington, H.B. & Briggs, D.E.G. (1985). The largest Cambrian animal *Anomalocaris*, Burgess Shale, British Columbia. *Philosophical Transactions of the Royal Society of London. Series B, Biological Sciences*, **309**:569—609.

Wright, K.A. (1987). The nematode’s cuticle: Its surface and the epidermis: function, homology, analogy: a current consensus. *The Journal of Parasitology*, **73**:1077—1083.

Yang, J., Ortega-Hernández, J., Gerber, S., Butterfield, N.J., Hou, J., Lan, T. & Zhang, X. (2015). A superarmoured lobopodian from the Cambrian of China and early disparity in the evolution of Onychophora. *Proceedings of the National Academy of Sciences*, **112**:8678—8683.

Zhang, X., Smith, M.R., Yang, J. & Hou, J. (2016). Onychophoran-like musculature in a phospahtized Cambrian lobopodian. *Biology Letters*, **12**:20160192.
