# Supplementary figures and images for: A three-dimensionally preserved lobopodian from the Herefordshire (Silurian) Lagerstätte, UK
Source: R Soc Open Sci. 2018 Aug 8;5(8):172101. doi: 10.1098/rsos.172101 (PMC6124121; doi:10.1098/rsos.172101)

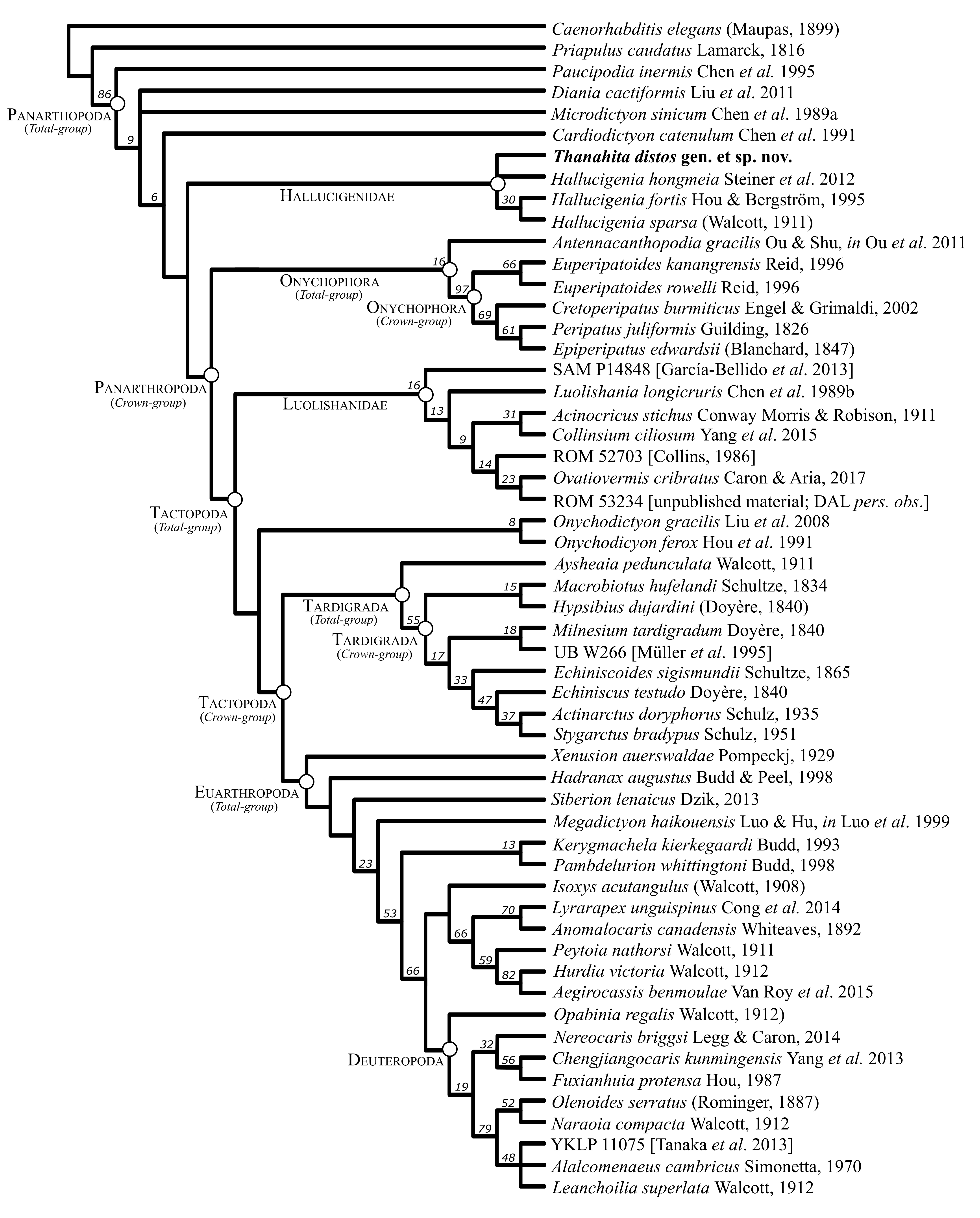

Supplement: ESM Figure S1 [file rsos172101supp2.png]
